# Metformin induces cell cycle arrest at the G1 phase through E2F8 suppression in lung cancer cells

**DOI:** 10.18632/oncotarget.21552

**Published:** 2017-10-06

**Authors:** Dong Hao Jin, Yujin Kim, Bo Bin Lee, Joungho Han, Hong Kwan Kim, Young Mog Shim, Duk-Hwan Kim

**Affiliations:** ^1^ Department of Molecular Cell Biology, Samsung Biomedical Research Institute, Sungkyunkwan University School of Medicine, Suwon, 440-746, Korea; ^2^ Department of Pathology, Samsung Medical Center, Sungkyunkwan University School of Medicine, Seoul, 135-710, Korea; ^3^ Department of Thoracic and Cardiovascular Surgery, Samsung Medical Center, Sungkyunkwan University School of Medicine, Seoul, 135-710, Korea

**Keywords:** metformin, lung cancer, cell cycle, E2F8, p21

## Abstract

A target molecule responsible for cell cycle arrest by metformin was discovered using a gene chip array in lung cancer cells and the effect of metformin on E2F8 was assessed. The siRNA-mediated knockdown of E2F8 significantly suppressed G1-S progression while ectopic expression of E2F8 relieved metformin-induced G1 arrest. The mRNA levels of p21 were found to be inversely related to those of E2F8 in lung cancer cells while siRNA-mediated knockdown of p21 partly rescued siE2F8-induced arrest of the cell cycle. Metformin had no effect on degradation of E2F8 mRNA. Activation and inhibition of AMPK by AICAR and Dorsomorphin, respectively, did not affect E2F8 suppression by metformin. The clinical significance of E2F8 was analyzed in The Cancer Genome Atlas (TCGA) data. One hundred six (13%) of 848 TCGA lung cancers overexpressed E2F8 mRNA. The overexpression of E2F8 was associated with poor overall survival (adjusted hazard ratio = 1.58, 95% confidence interval = 1.13–2.22; P = 0.008). The present study suggests that metformin may induce cell cycle arrest at the G1 phase by suppressing E2F8 expression in lung cancer cells. In addition, E2F8 may be associated with poor overall survival in lung cancer patients irrespective of histology.

## INTRODUCTION

Lung cancer is the leading cause of cancer-related death in the world. Despite recent advances in molecular target therapy, the cure rate of advanced lung cancer remains low. Recently, oral antidiabetic drug metformin has emerged as an attractive agent for lung cancer therapy. Metformin is associated with improved overall survival in patients with diabetes with stage IV non- small cell lung cancer (NSCLC) [1]. Combination therapy of metformin with anti-IGF1R mAb Figitumumab or an epidermal growth factor receptor (EGFR) tyrosine kinase inhibitor produces a synergistic effect in small cell lung cancer (SCLC) and NSCLC [2–4]. The anticancer effect of metformin is thought to be partly due to inhibition of cell proliferation [5–7]. Metformin blocks the cell cycle in the G1 phase through upregulation of p21 [8] or downregulation of cyclin D1 and E2F1 expression in prostate, breast, and bladder cancer cells [9–11]. However, cyclin D1 expression was not affected by metformin in lung cancer cells while E2F1 knockout cells showed normal proliferation in a mouse model [12, 13], implying that other factors may be involved in metformin-induced cell cycle arrest in lung cancer cells.

The E2F family is a core transcriptional axis crucial for cell cycle transitions by regulating gene expression, including expression of cyclins and CDKs. Traditionally, E2Fs are categorized into three groups based on their transcriptional activity: activators (E2F1-E2F3), canonical repressors (E2F4-E2F6), and atypical repressors (E2F7-E2F8). Upon mitogenic stimulation, activated E2F1-E2F3 will accumulate in the late G1 phase and initiate a transcriptional program that drives cells into S phase. The G1/S-specific transcriptome is then attenuated by the action of E2F7 and E2F8 [14–16]. However, recent studies have revealed that atypical repressors have a role in cell cycle promotion. Expression of E2F7 and E2F8 is tightly correlated with expression of proliferative marker Ki-67 and associated with hepatocyte proliferation [17]. Upregulation of E2F8 promotes cell proliferation and tumorigenicity in breast, hepatocellular, and lung cancers [18–20]. E2F8 accelerates the S-phase transition by transcriptionally upregulating cyclin E1 and cyclin E2 in breast cancer cells [18] and cyclin D1 in hepatocellular cellular carcinoma [19] through interactions with regulatory elements in their promoters. E2F7 and E2F8 form homodimers (E2F7-E2F7 and E2F8-E2F8) or heterodimers (E2F7-E2F8) to control transcription of cell cycle-related genes [14, 21, 22], and both atypical (E2F7, E2F8) and typical (E2F1-E2F3) E2Fs bind to similar DNA sequences [23].

In this study, we analyzed global gene expression in metformin-treated lung cancer cells and found that E2F8 was significantly downregulated by metformin. The expression levels of E2F8 were inversely correlated to those of p21, and metformin-induced overexpression of p21 was independent of p53. In addition, E2F8 overexpression was found to be significantly associated with poor overall survival in lung cancer.

## RESULTS

### Metformin induces cell cycle arrest at the G1 phase in lung cancer cells

**Figure 1:**
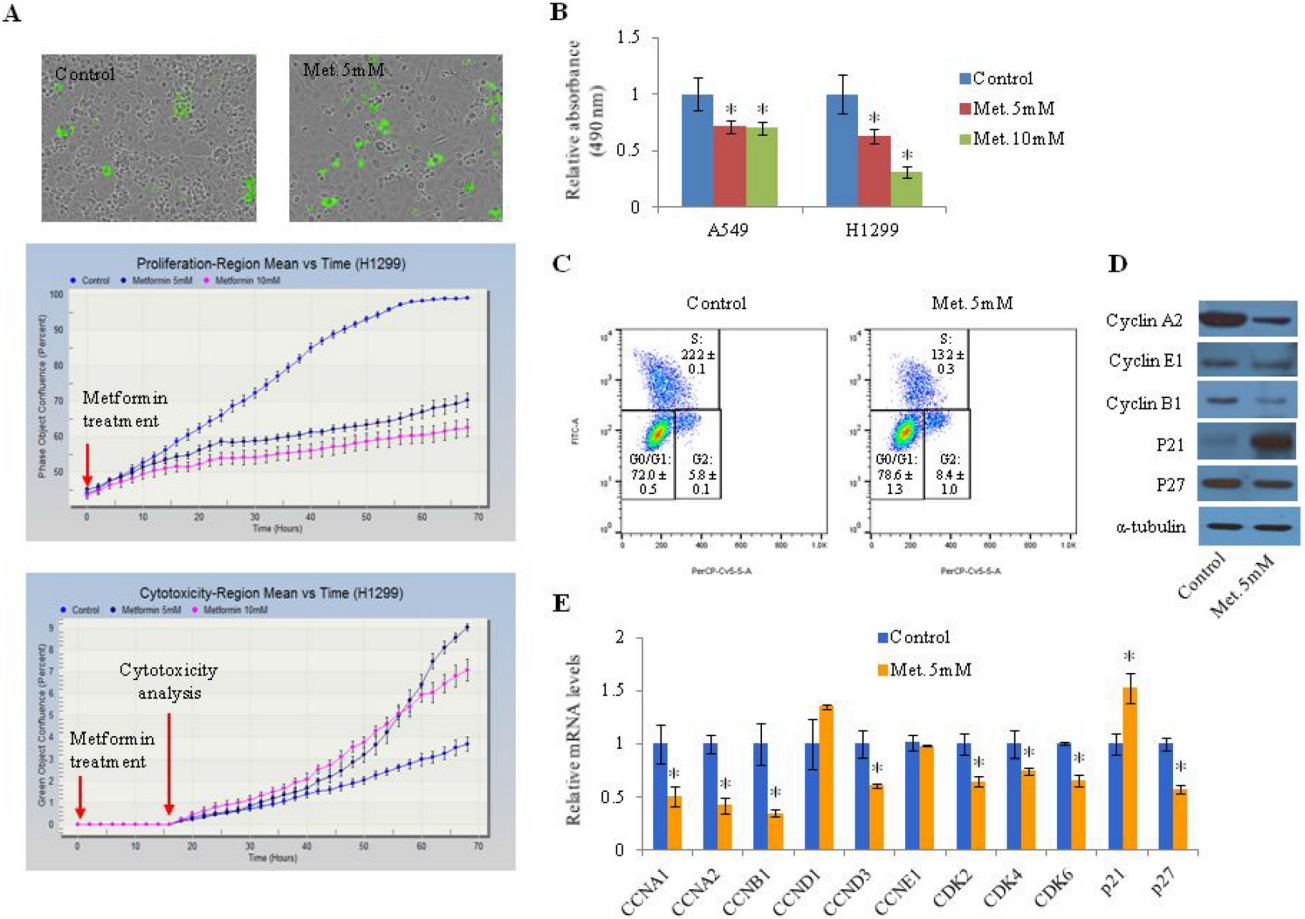
Effect of metformin on proliferation of lung cancer cells (**A**) H1299 cells were treated with metformin (5 mM or 10 mM). Upper panels: representative images of cultured H1299 cells. Green indicates dead cells stained with cyanine dye. Middle panel: percentage of confluence. The images were obtained every two hours and the percent confluence was calculated. Lower panel: percentage of regions displaying cytotoxicity. The cyanine dye was added 16 h after metformin treatment. (**B**) Cell proliferation was detected by MTS assay. Error bars indicate standard deviation (*n* = 8, ^*^P < 0.05). (**C**) H1299 cells were treated with BrdU and labeled with a FITC-conjugated anti-BrdU antibody. Total DNA was stained with 7-AAD and the percentage of cells in each stage of the cell cycle was analyzed. This experiment was performed three times and similar results were obtained each time. (**D** and **E**) H1299 cells were treated with 5 mM metformin for 48 h and the protein (D) and mRNA (E) levels of cell cycle-related genes were measured by western blotting and qRT-PCR, respectively. Relative mRNA levels indicate fold change in mRNA levels of metformin-treated cells compared to control. Error bars indicate standard deviation (*n* = 3, ^*^P < 0.05). “met.” indicates metformin.

To investigate the effect of metformin on cell proliferation and cell cycle as well as the expression of several cell cycle-related genes in lung cancer cells, H1299 (Figure 1A) and A549 cells (Supplementary Figure 1A) were treated with 5 mM or 10 mM metformin. Metformin significantly inhibited cell proliferation in a dose-dependent manner and increased the proportion of dead cells (Figure 1A, Supplementary Figure 1A). The anti-proliferative effect of metformin was apparent at 14 h after treatment in H1299 (Figure 1A) and A549 cells (Supplementary Figure 1A). The induction of cell death by metformin was observed at 44 h and 30 h post-treatment in H1299 (Figure 1A) and A549 cells (Supplementary Figure 1A), respectively. Metformin-induced inhibition of cell proliferation was measured by MTS assay (Figure 1B). Metformin was found to arrest the cell cycle at G1 phase in lung cancer cells: the proportion of G0/G1 phase cells was increased from 72.3% to 79.5% after treatment with metformin (Figure 1C). Cell cycle-related genes such as cyclin A1, cyclin A2, cyclin B1, cyclin D3, CDK2, CDK4, and CDK6 were downregulated in response to metformin while p21 was upregulated by metformin (Figure 1D and 1E, Supplementary Figure 1B).

### E2F8 mediates metformin-induced cell cycle arrest in lung cancer cells

**Figure 2:**
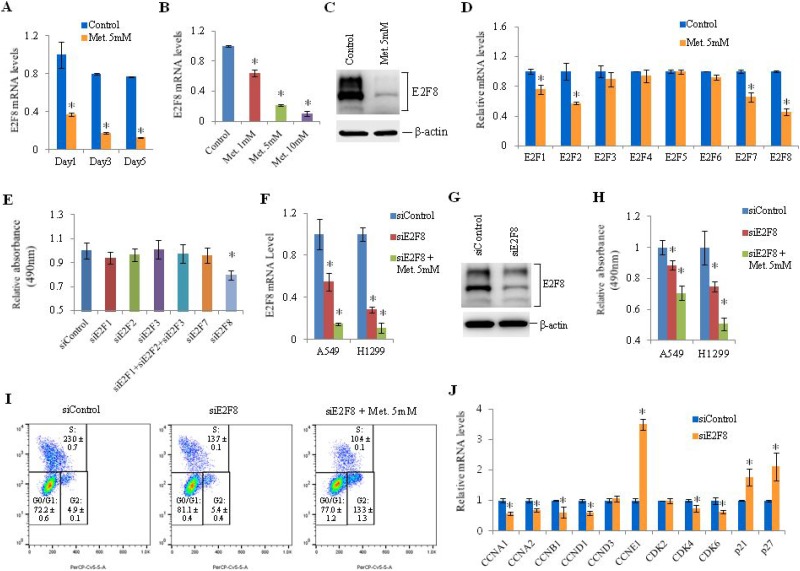
Effect of metformin on E2F8 expression and effect of E2F8 knockdown on proliferation of lung cancer cells (**A**) H1299 cells were treated with 5 mM metformin and E2F8 mRNA levels were measured by qRT-PCR. RPLP0 was used as an internal control. Relative E2F8 mRNA levels were calculated by comparing it to the expression level of the control. Error bars indicate standard deviation (*n* = 3, ^*^P < 0.05). (**B**) H1299 cells were treated with metformin (1 mM, 5 mM, 10 mM), and E2F8 mRNA levels were measured by qRT-PCR (*n* = 3, ^*^P < 0.05). (**C**) E2F8 and β-actin protein levels were analyzed by western blot. Experiments were performed three times and similar results were obtained each time. (**D**) The mRNA levels of the eight E2F family members were measured by qRT-PCR in H1299 cells exposed to 5 mM metformin for 48 h (*n* = 3, ^*^P < 0.05). (**E**) H1299 cells were transfected with the indicated siRNAs and cell proliferation was analyzed using MTS assay on the third day after transfection (*n* = 8). (**F**) A549 and H1299 cells were transfected with an off-target control siRNA (siControl), siE2F8, or siE2F8 plus 5 mM metformin, and relative mRNA levels of E2F8 normalized to RPLP0 were measured by qRT-PCR (*n* = 3, ^*^P < 0.05). (**G**) E2F8 protein levels in H1299 cells were analyzed using western blot. (**H**) Cell proliferation was measured by MTS assay on the third day after treatment with siE2F8 and/or 5 mM metformin (*n* = 8). (**I**) H1299 cells were treated with BrdU and labeled with a FITC-conjugated anti-BrdU antibody. Total DNA was stained with 7-AAD. The percentage of cells in each stage (G0/G1, S-phase, G2) of the cell cycle was measured after transfecting siE2F8. This experiment was performed three times and similar results were obtained each time. (**J**) The mRNA levels of cell cycle-related genes were measured by qRT-PCR after transfection of siE2F8. Values on the y-axis are expressed as fold change in mRNA levels relative to control (*n* = 3, ^*^P < 0.05).

To find novel targets involved in metformin-induced cell cycle arrest in lung cancer cells, we analyzed mRNA levels using GeneChipÒ Human Gene ST Arrays in A549 cells treated with metformin. Genes that were 1.5 fold up- or down- regulated compared to the control were classified using DAVID (The Database for Annotation, Visualization and Integrated Discovery) (Supplementary Tables 4–7) [24]. Apoptosis-related genes such as CHAC1, DDIT4, TRIB3, TP53INP1, and TP63 were up-regulated while cell cycle-related genes such as E2F8, CCNF, CCND3, CCNB3 and CDC6 were down-regulated by metformin treatment. Among cell cycle-related genes, E2F8 was the most prominently down-regulated (Log2 Ratio = –0.9603) by metformin (Supplementary Table 7). Metformin inhibited mRNA expression of E2F8 in various lung cancer cell lines (H23, H226, A549, and H1299) (Supplementary Figure 2A). The inhibitory effect of metformin on E2F8 expression occurred in a dose- and time-dependent manner in H1299 cells (Figure 2A and 2B). E2F8 expression was also inhibited by metformin in H1299 cells (Figure 2C). Among the eight members of the E2F family, metformin suppressed mRNA expression of E2F1, E2F2, E2F7, and E2F8 (Figure 2D, Supplementary Figure 2B) while E2F8 was most significantly associated with cell proliferation (Figure 2E, Supplementary Figure 2C). The addition of metformin to E2F8-knockdown H1299 cells suppressed E2F8 expression (Figure 2F and 2G) and inhibited cell proliferation (Figure 2H) and G1/S progression (Figure 2I) synergistically. The proportion of cells in S phase was decreased from 22.5% to 13.7% by siRNA-mediated knockdown of E2F8. It was further reduced to 10.3% by addition of metformin (Figure 2I). These observations suggest that metformin may be involved in E2F8 suppression and cell cycle arrest via a mechanism that does not involve siRNA. To investigate downstream target proteins of E2F8, we knocked it down in H1299 cells using siE2F8 and analyzed mRNA levels of cell cycle-related genes using qRT-PCR. Cyclin A1, cyclin A2, cyclin B1, cyclin D1, CDK4, and CDK6 were down-regulated while p21 and p27 were up-regulated (Figure 2J).

### E2F8 regulates cell proliferation partly through control of p21 expression in lung cancer cells

**Figure 3:**
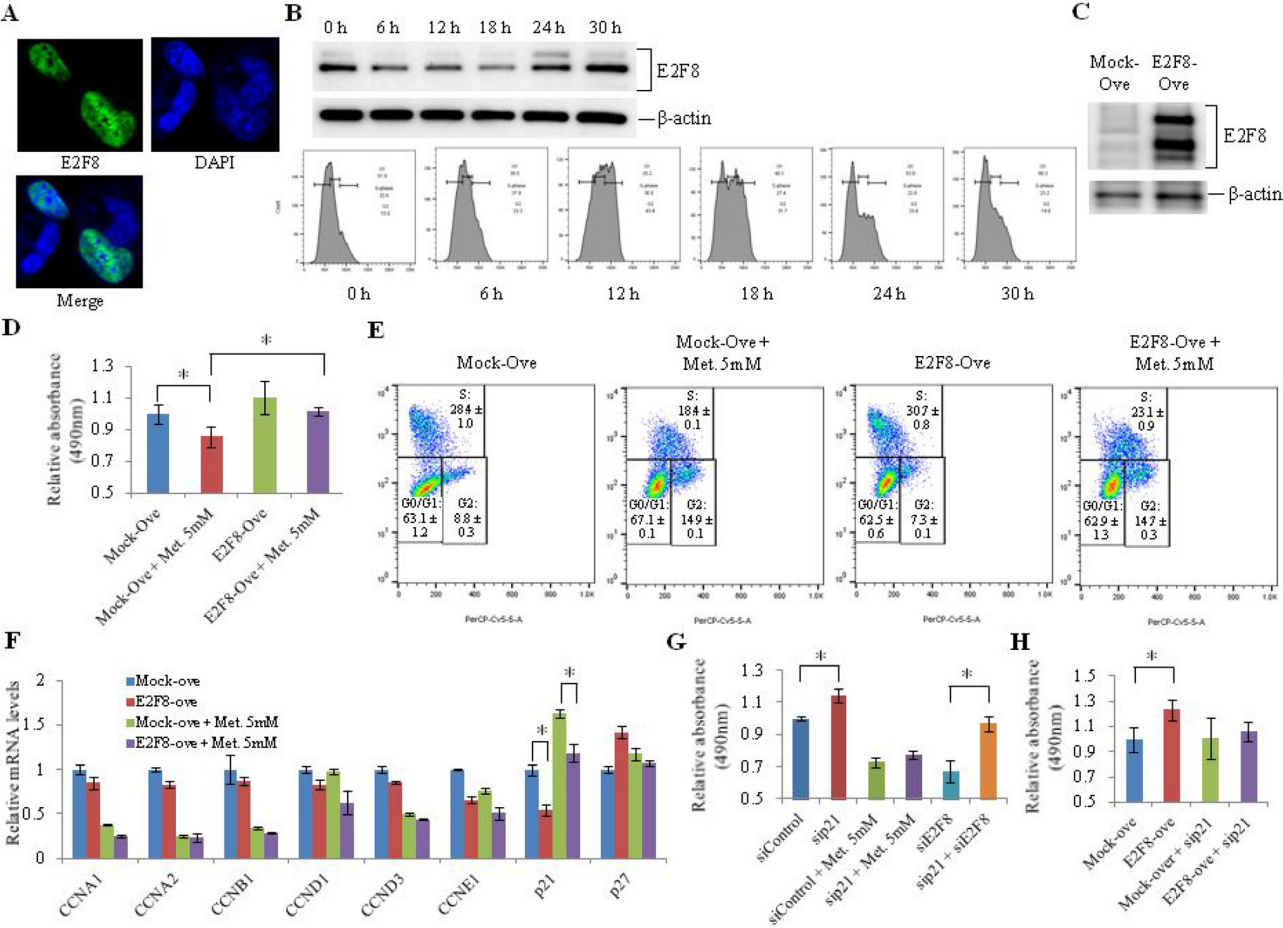
Effect of E2F8 overexpression on proliferation of lung cancer cells (**A**) H1299 cells were transfected with the pCMV6-Entry vector expressing GFP-tagged human E2F8 and images were obtained by immunofluorescence microscopy. DNA was stained with DAPI. (**B**) After 24 h of hydroxyurea treatment, E2F8 protein levels (upper panel) and cell cycle (lower panel) were analyzed using western blotting and FACS with PI staining, respectively, at 6, 12, 18, 24, and 30 h. (**C**) E2F8 expression was verified using western blot analysis in H1299 cells transfected with an E2F8 expression vector. (**D**–**F**) A plasmid encoding E2F8 was transfected into H1299 cells and incubated with or without 5 mM metformin. Cell proliferation (D) and cell cycle (E) were analyzed using MTS assay and FACS on the third day after transfection (*n* = 8, ^*^P < 0.05). The mRNA levels of cell cycle-related genes (F) were also measured by qRT-PCR (*n* = 3, ^*^P < 0.05). (**G** and **H**) H1299 cells were transfected with siRNA directed against p21 or E2F8 as well as with E2F8 expression plasmid. Cell proliferation was then analyzed using MTS assay on the third day after transfection (*n* = 8, ^*^P < 0.05). “Ove” represents overexpression.

To further demonstrate the inhibitory effect of metformin on cell proliferation through E2F8 and p21, we overexpressed E2F8 in H1299 cells and treated cells with metformin. We first analyzed E2F8 expression during cell cycle progression. Like other E2F family members, E2F8 was principally expressed in the nucleus (Figure 3A) where it exerted its role as a transcription factor. E2F8 protein levels were low at 6 h, 12 h, and 18 h into the cell cycle, with a low G1 phase population. However, they were upregulated at 24 h and 30 h with an increase in G1 phase population, suggesting that E2F8 was activated in G1 phase and downregulated in S and G2 phases (Figure 3B). Overexpression (Figure 3C) of E2F8 by transient transfection in H1299 cells increased cell proliferation (Figure 3D) and G1-S progression of the cell cycle (Figure 3E). The addition of metformin to E2F8-overexpressing cells resulted in a decrease in cell proliferation and G1-S progression (Figure 3E). The proportion of S phase cells, which was increased by overexpression of E2F8, was decreased from 30.1% to 23.7% following the addition of metformin (Figure 3E). Among cell cycle related-genes such as cyclin A1, cyclin A2, cyclin B1, cyclin D1, cyclin D3, and cyclin E1, only p21 was significantly downregulated by E2F8 overexpression in H1299 cells. The addition of metformin resulted in the recovery of p21 expression (Figure 3F). To understand the contribution of p21 to inhibition of cell proliferation by metformin through E2F8 suppression, we analyzed cell proliferation in p21-knockdown H1299 cells (Figure 3G and 3H). Cell proliferation was increased in H1299 cells treated with sip21 but decreased in those treated with 5 mM metformin or siE2F8. However, in p21-knockdown H1299 cells, the addition of siE2F8 had a minimal effect on cell proliferation (Figure 3G). In addition, E2F8 overexpression increased cell proliferation in H1299 cells. However, its effect was minimal in p21-knockdown cells.

### Metformin-induced E2F8 suppression is not dependent on AMPK activation

**Figure 4:**
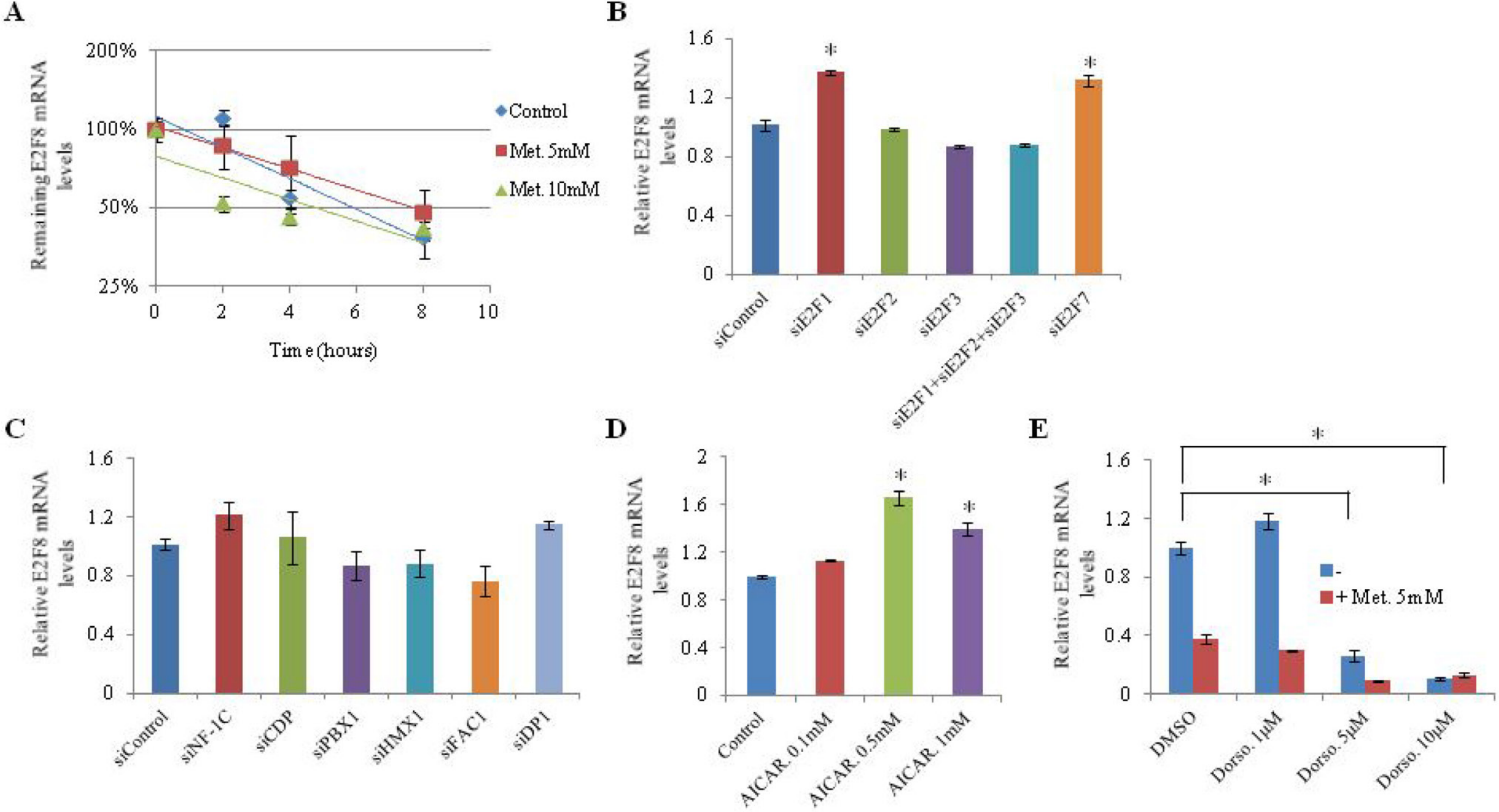
Effect of transcription factors and AMPK on E2F8 expression (**A**) H1299 cells were treated with metformin (5 mM, 10 mM). After inhibition of *de novo* mRNA synthesis with actinomycin D, E2F8 mRNA levels were measured by qRT-PCR at indicated time points. Error bars indicate one standard deviation (*n* = 3, ^*^P < 0.05). (**B**–**E**) H1299 cells were transfected with the indicated siRNAs directed against E2Fs (B) or transcription factors (C) and incubated with AMPK activator AICAR (D) or AMPK inhibitor dorsomorphin (Dorso) (E) with or without 5 mM metformin. E2F8 mRNA levels were measured by qRT-PCR (*n* = 3, ^*^P < 0.05). The values on the y-axis represent fold change in E2F8 mRNA level relative to the control (*n* = 3, ^*^P = 0.05). Error bars indicate one standard deviation in the fold change of E2F8 mRNA levels.

To investigate the effect of metformin on E2F8 mRNA stability, we analyzed the rate of E2F8 mRNA degradation in H1299 cells. The mRNA level of E2F8 was reduced to half within 4–8 hours after inhibition of *de novo* mRNA synthesis by actinomycin D. However, no difference in mRNA level of E2F8 was observed between control and metformin-treated cells (Figure 4A). A previous study has shown that E2F1 binds to the E2F8 promoter region and controls its mRNA expression [21]. Therefore, we analyzed the influence of E2F1, E2F2, E2F3, E2F7, and E2F8 on each other's expression by knocking down individual E2Fs using siRNA in H1299 cells (Supplementary Figure 3). No suppressive effect on E2F8 expression was observed (Figure 4B). To further understand the underlying mechanism by which metformin suppresses E2F8, we searched for transcription factors downregulated by metformin in the microarray data and found candidate transcription factors, including NF-1, E2F, DP1, CDP, PBX1, HMX1, and FAC1 (Supplementary Table 8). However, siRNA-medicated knockdown of these factors did not affect the expression of E2F8 (Figure 4C). Metformin is known to exert its actions in part through AMPK activation. However, alteration of AMPK activity by AICAR or dorsomorphin did not affect the mRNA level of E2F8 in H1299 cells (Figure 4D and 4E), suggesting that metformin-induced E2F8 suppression may not be dependent on AMPK activity. Either AICAR or Dorsomorphin treatment suppressed A549 and H1299 cells proliferation (Supplementary Figure 4).

### Overexpression of E2F8 is associated with poor overall survival in patients with non-small cell lung cancer

**Figure 5:**
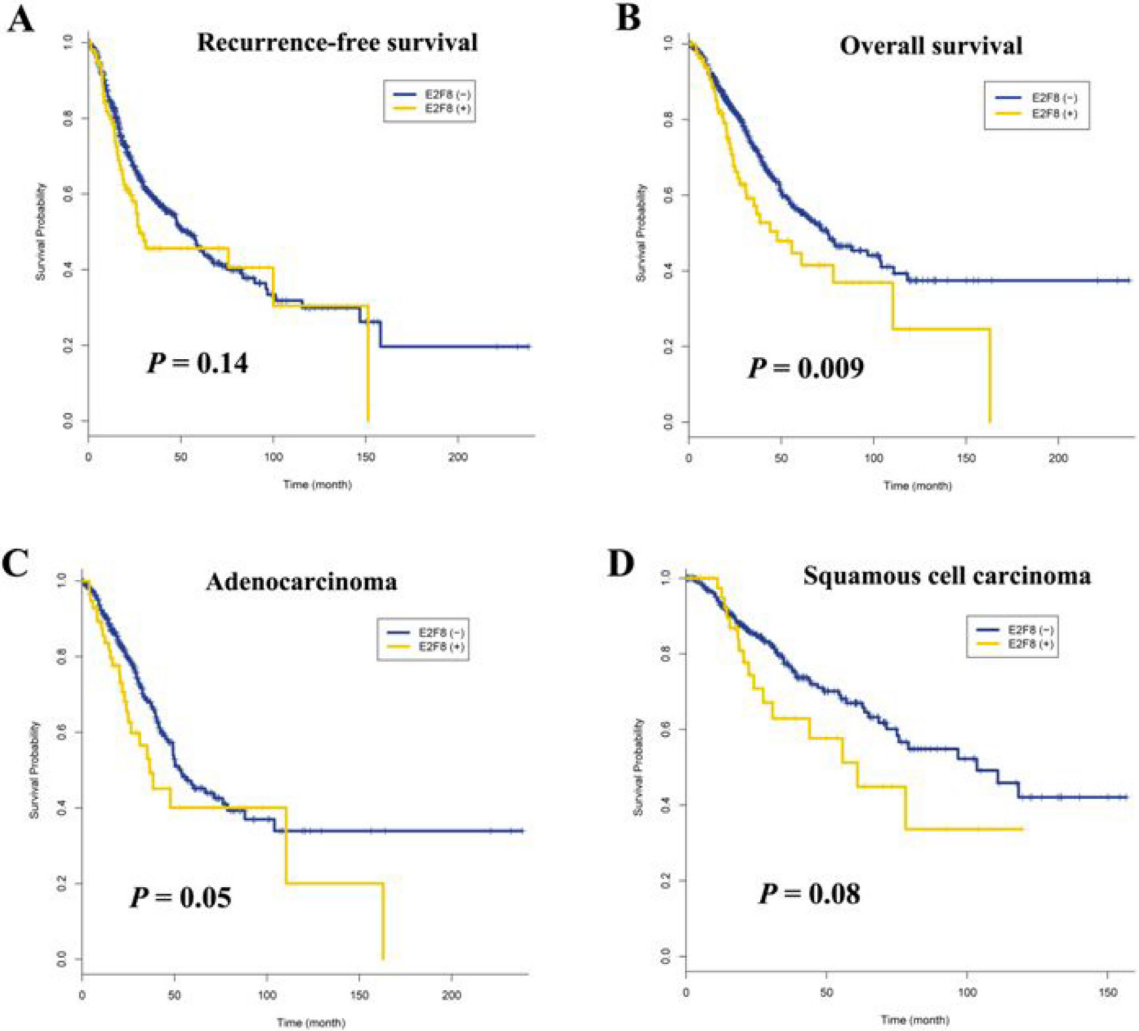
Effect of E2F8 overexpression on survival of patients with non-small cell lung cancer (**A**) Recurrence-free survival was not associated with E2F8 overexpression (*P* = 0.14). (**B**) The five-year overall survival rate was 34% and 46% in patients with and without E2F8 overexpression, respectively. This difference was statistically significant (*P* = 0.009). (**C** and **D**) Data were stratified according to histology and overall survival was analyzed in 475 adenocarcinomas (C) and 373 squamous cell carcinomas (D). The *P*-values are based on log-rank test.

The effect of E2F8 overexpression on patient survival was analyzed using TCGA (The Cancer Genome Atlas) lung cancer data. The clinicopathological characteristics of 848 participants are described in Supplementary Table 9. Overexpression of E2F8 was defined as a z-score greater than or equal to one. It was found in 58 (12%) of 475 adenocarcinomas and 48 (13%) of 373 squamous cell carcinomas. Overall survival and recurrence-free survival (RFS) were compared between patients with and without E2F8 overexpression. Recurrence-free survival was not significantly different between patients with E2F8 overexpression and those without (*P* = 0.14; Figure 5A), but overall survival was poorer in patients with E2F8 overexpression than in those without (*P* = 0.009; Figure 5B). In addition, the effect of E2F8 overexpression on overall survival was similar in adenocarcinoma (*P* = 0.05; Figure 5C) and squamous cell carcinoma (*P* = 0.08; Figure 5D). Overall survival in patients with E2F8 overexpression was 1.58 (95% confidence interval = 1.13–2.22, *P* = 0.008) times poorer than that in patients without E2F8 overexpression after adjusting for pathologic stage, age, histology, and recurrence (Table 1). However, RFS was not associated with E2F8 overexpression.

**Table 1:** Cox proportional hazards analysis of survival in 848 NSCLCs

	E2F8 overexpression	HR^a^	95% CI^a^	*P*-value
Overall survival^b^	No	1.00		
	Yes	1.58	1.13–2.22	0.008
RFS^c^	No	1.00		
	Yes	1.26	0.92–1.72	0.15

^a^Abbreviations: HR, hazard ratio; CI, confidence interval; RFS, recurrence-free survival.

^b^Adjusted for recurrence, pathologic stage, histology, and age.

^c^Adjusted for histology and pathologic stage.

## DISCUSSION

Metformin exerts anticancer effects mainly by suppressing cell proliferation and inducing cell death in lung cancer cells. To understand the molecular mechanisms underlying metformin-induced inhibition of cell proliferation, we analyzed expression changes in mRNA levels using the Affymetrix GeneChip® Human Gene 1.0 ST Array. Metformin down-regulated the expression of cell cycle-related genes such as E2F8, CCNF, CCND3, CCNB3, and CDC6 but up-regulated the expression of apoptosis-related genes such as CHAC1, DDIT4, TRIB3, TP53INP1, and TP63. Among them, we focused on E2F8 because of its dramatic down-regulation by metformin. Metformin down-regulated four E2F family members, E2F1, E2F2, E2F7, and E2F8 in lung cancer cells, with E2F8 being the most prominently down-regulated one (Supplementary Table 7). In this study, siRNA-mediated knockdown of E2F8 in lung cancer cells significantly suppressed cell proliferation while overexpression of E2F8 recovered metformin-induced inhibition of cell proliferation, suggesting that E2F8 may be a molecular target for inhibition of proliferation by metformin in lung cancer cells.

It is currently unclear what is responsible for the extra bands shown in Figure 3. The rabbit polyclonal anti-E2F8 antibody (Abcam, ab109596) used in this study is known to react specifically with mouse and human E2F8. This antibody is known to produce only one band in HeLa cells. All bands appeared in H1229 cells transfected with E2F8 expression plasmid, but not in control cells (Figure 3C). There is no report about isoforms of E2F8 in human cells. One possibility is that E2F8 might have undergone posttranslational modification such as phosphorylation in H1299 cells. Different intracellular environments of HeLa cells and H1229 cells might affect posttranslational modification. E2F family members are frequently deregulated in lung cancer. E2F1, E2F2, and E2F3 are up-regulated in non-small cell lung cancer. Overall survival in lung cancer patient is known to be poorer in patients with over-expression of E2F1 or E2F2 than in those with normal expression [25–27]. E2F8 is also up-regulated in lung cancer. Its over-expression is associated with poor prognosis [20]. The present study also observed worse overall survival in lung cancer patients with over-expression of E2F8 than in those with normal E2F8 expression, irrespective of histology. These observations suggest that E2F8, in addition to E2F1 and E2F2, could be useful as a prognostic biomarker in lung cancer.

E2F8 is known as a repressor of E2F activators such as E2F1 [28]. During the cell cycle, accumulation of newly synthesized E2F1, E2F2, and E2F3 in late G1 phase initiates a transcriptional program that drives G1 phase cells into the S phase. This G1-S-specific transcriptome is then attenuated by the action of repressors E2F7 and E2F8 [14]. Based on this scenario, suppression of E2F8 will release a brake on E2F1 and continue the activation of E2F1 target genes, resulting in ectopic DNA replication. E2F1 overexpression in quiescent fibroblasts leads to induction of cellular DNA synthesis and apoptosis [29]. Knockdown of E2F8 in lung cancer significantly increases the proportion of dead A549 cells in culture [20]. The upregulation of E2F1 target genes such as cyclin E1 (Figure 2J), PCNA, and MCM3 by E2F8 knockdown of E2F8 supplementary Table S6 in this study may support this scenario. However, considering the suppression of DNA synthesis after E2F8 knockdown observed in this study, it is possible that E2F8 may play a role in cell cycle progression through other mechanisms in addition to E2F1. Park et al. [20] have searched for E2F8-recognizing DNA sequences and identified the most highly represented motifs recognized by transcription factors such as E2F1, E2F4, E2F6, E2F7, NFY, Elk1, Elk4, and Sp1. This suggests that transcriptional activities of NFY, Elk1, and Sp1 may also be suppressed by E2F8.

The anticancer effect of metformin is known to involve up-regulation of p21 [8]. In the present study, p21 expression was inversely correlated with E2F8 expression, suggesting that E2F8 might be a transcriptional repressor of p21. It is unlikely that E2F8 decreases p21 expression by suppressing the transcriptional activity of p53 because the E2F8-p21 relationship is detected in a p53-null lung cancer cell line, H1299. The p53-independent transactivation of *p21* by activated Ras requires transcription factor E2F1 [30] while E2F1 and E2F3 strongly activate *p21* transcription by binding to *cis*-acting elements of p21 [31], which may suggest that E2F8 negatively regulates p21 by suppressing E2F1 or E2F3. Many transcription factors such as SP1, SP3, AP2, CCAAT/enhancer binding protein-α (C/EBPα), C/EBPβ, BETA2 (also known as NEUROD1), GAX (also known as MOX2), homeobox A10 (HOXA10), STATs, and myoblast determination protein 1 (MYOD1) control *p21* transcription in a p53-independent manner [32]. Therefore, negative regulation of p21 expression by E2F8 may be accomplished by suppressing these transcription factors. Motif analysis has revealed that Sp1 binding site is one of the top E2F8 target DNA sequences [20]. Whether E2F8 directly binds to the p21 promoter region and whether transcription factors that induce p21 expression are suppressed by E2F8 are topics that require further study.

The mechanism underlying E2F8 downregulation by metformin was investigated in this study. Metformin controlled E2F8 expression at the transcription level. We investigated some possible TFs such as NF-1, E2F1, E2F2, E2F3, E2F7, DP1, CDP, PBX1, HMX1, and FAC1. However, none of them was the candidate. A previous study has shown that Naphthol AS-TR phosphate (NASTRp), an inhibitor of cAMP response element-binding protein (CREB) transcriptional activity, significantly suppresses E2F8 expression in lung cancer [20]. Therefore, it is possible that metformin regulates E2F8 through this pathway because metformin has been shown to suppress cAMP/CREB signaling through phosphorylation of CREB binding protein (CBP) in hepatoma cells [33]. Interestingly, in the present study, dorsomorphin significantly suppressed E2F8 expression and the effect was not AMPK dependent. Dorsomorphin has been shown to inhibit BMP-mediated Smad, p38, and Akt signaling [34]. We speculate that some of these pathways may be critical to the regulation of E2F8 expression in lung cancer. We are still investigating the mechanisms by which metformin modulates E2F8 expression and its anticancer effects.

## MATERIALS AND METHODS

### Cell culture and counting

Lung cancer cell lines H23, H226, H460, A549, H1299, and H1650 were obtained from the American Type Culture Collection (ATCC). Characterizations of these cell lines are available at http://cellbank.snu.ac.kr. These cells were cultured in RPMI 1640 medium (Lonza, Allendale, NJ) supplemented with 10% fetal bovine serum (FBS), 100 U/mL penicillin, and 100 μg/mL streptomycin at 37°C in a humidified atmosphere with 5% CO_2_. Cells were treated with different concentrations of metformin (1, 5, and 10 mM) (D150959, Sigma) and cell growth was measured by a real-time quantitative live-cell analysis system, IncuCyte^®^ ZOOM System (Essen BioScience, Ann Arbor, MI, USA) [35]. Briefly, cells were seeded into a 24-well plate at a density of 1 × 10^5^ cells per well and phase-contrast images were obtained at each time point. Data were analyzed using the IncuCyte software according to the manufacturer's instructions. Dead cells were counted using cyanine dye (G8741, Promega, Madison, WI, USA).

### MTS assay

Cells were cultured in 96-well plates (8000 cells/well) and transfected with siRNA (40 nM) or the pCMV6-Entry vector (0.6 μg/ml). Cell proliferation was measured using the MTS [3-(4,5-dimethylthiazol-2yl)-5-(3-carboxymethoxyphenyl)-2-(4-sulphophenyl)-2H-tetrazolium] assay on the third day after transfection as described previously [36]. CellTiter 96 Aqueous One Solution (Promega, Madison, WI) was added to each well and cells were incubated for 1 hour in a 37°C incubator with 5% CO_2_. Absorbance was measured at 490 nm using a microplate reader (Bio-Rad, Hercules, CA, USA).

### Microarray

A549 cells were cultured in RPMI 1640 medium supplemented with 5 mM metformin for 48 h. Total RNA was isolated from these cells using the PureLink RNA Mini Kit (Invitrogen, Carlsbad, CA, USA). Double-stranded cDNA was synthesized from total RNA and *in vitro* transcription was performed to produce biotin-labeled cRNA using GeneChip One-Cycle Target Labeling and Control Reagents (Affymetrix, Santa Clara, CA, USA) according to the manufacturer's instructions. After fragmentation, the cRNA was hybridized with the Affymetrix GeneChip^®^ Human Gene 1.0 ST Array (Affymetrix). GeneChips were then scanned in a GeneChip Scanner 3000 (Affymetrix). Normalization, filtering, and gene expression analysis of the data were performed with the Affymetrix GCOS software.

### Small interfering RNA (siRNA)-mediated gene silencing and plasmid-mediated gene overexpression

To knockdown target genes, cells were transiently transfected with 40 nM of gene-specific siRNA (BioNeer, DaeJeon, Korea) or non-targeting siRNA (BioNeer) as a negative control. The siRNA sequences used for knockdown experiment are shown in Supplementary Table 1.

The pCMV6-Entry vector expressing GFP-tagged human E2F8 (OriGene, Rockville, MD, USA) was used to analyze gene expression and Lipofectamine 2000 (Invitrogen, Carlsbad, CA, USA) was used to transfect siRNA or plasmid into cells according to the manufacturer's protocol. At 48 h post-transfection, gene expression was measured using quantitative real-time PCR or western blot analysis.

### Quantitative real-time PCR

mRNA level was analyzed by quantitative real-time PCR. Total RNA was isolated using the PureLink RNA Mini Kit (Invitrogen, Carlsbad, CA, USA) and reverse transcription was carried out using the SuperScript VILO cDNA Synthesis Kit (Invitrogen, Carlsbad, CA, USA). Quantitative real-time PCR was performed with SYBR green dye (4385614, Applied Biosystems, Foster City, CA, USA) using the ABI PRISM 7900HT Sequence Detection System (Applied Biosystems) under the following conditions: initial denaturation for 5 min at 95°C, followed by 40 cycles of 5 s at 95°C and 30 s at 60°C. PCR primers were designed using Primer Express 3 (Applied Biosystems, Foster City, CA, USA) and their specificity was checked by BLAST analysis. PCR primer sequences used for qRT-PCR are listed in Supplementary Table 2.

### Western blot analysis

Total proteins were extracted from cultured cells using a lysis buffer containing protease inhibitor cocktail (Roche Applied Science, Indianapolis, IN, USA). Cell lysates were heated at 95°C for 5 min, loaded on 10% sodium dodecyl sulfate-polyacrylamide gels, and transferred to a PVDF membrane (Immobilon-P, Millipore, Medford, MA, USA). After blocking with a 3% solution of fetal bovine serum, membranes were probed with antibodies (listed in Supplementary Table 3). These membranes were then incubated with horseradish peroxidase-conjugated secondary antibodies (Cell Signaling Technology) and visualized with the Immun-Star Western Kit (Bio-Rad, Hercules, CA, USA).

### Immunocytochemistry

To observe E2F8 expression in cells, H1299 cells were transfected with the pCMV6-Entry vector expressing GFP-tagged human E2F8. Cells were fixed with 4% PFA and images were obtained by a immunofluorescence microscope Zeiss AX10 (Zeiss, Gottingen, Germany). DNA in cells was stained with DAPI.

### Cell cycle analysis using fluorescence-activated cell sorting (FACS)

The cell cycle was analyzed using the FITC BrdU Flow Kit (#559619, BD, Franklin Lakes, NJ, USA) or propidium iodide (PI) solution (50 μg/mL) according to the manufacturer's protocol. Briefly, cells were cultured in 6-well plates and transfected with siRNA or plasmid vectors. On the third day post-transfection, cells were incubated with 10 μM BrdU for 2 hours before harvest. Cells were resuspended, washed twice in phosphate-buffered saline (PBS), and fixed. DNA was stained with 7-AAD or PI and then analyzed using a FACS Calibur system (BD, Franklin Lakes, NJ, USA) and CELLQuest software (version 3.3; Becton Dickinson).

### Transcription factor binding site (TFBS) analysis

The transcription factors (TFs) responsible for downregulation of genes (<1.5) by metformin were analyzed using TRANSFAC and ExPlain (Qiagen, Germany). TRANSFAC and ExPlain use F-Match to search for putative TFBSs and TFs by a weight matrix-based algorithm through comparing a Yes-set (experimental set) to a No-set (control or background set) [37]. Promoter regions from -2000 to +100 bp around the transcription start site of downregulated genes (Yes-set) were selected. The degree of enrichment was calculated against a random of housekeeping genes (No-set) using a cut-off value to minimize false positives and a *p*-value threshold of > 0.01.

### Statistical analysis

The effect of E2F8 overexpression on survival was estimated by Kaplan–Meier survival curves and the significance of differences in survival between two groups was evaluated by the log-rank test. Cox proportional hazards analysis was performed to estimate hazard ratios of independent factors for survival after controlling for potential confounding factors. All statistical analyses were two-sided with a 5% type I error rate.

## SUPPLEMENTARY MATERIALS FIGURES AND TABLES



## Abbreviations

SCLC: small cell lung cancer
NSCLC: non small cell lung cancer
CDK: cyclin-dependent kinase
qRT-PCR: quantitative real-time PCR
E2F8: E2F transcription factor 8
AMPK: AMP-activated protein kinase
TFBS: transcription factor binding site
