# Supplementary material for: Metformin induces cell cycle arrest at the G1 phase through E2F8 suppression in lung cancer cells
Source: Oncotarget. 2017 Oct 6;8(60):101509–19. doi: 10.18632/oncotarget.21552 (PMC5731892; doi:10.18632/oncotarget.21552)
Supplement: Supplementary file 1 [file oncotarget-08-101509-s001.pdf]

Metformin induces cell cycle arrest at the G1 phase through E2F8 suppression in lung cancer cells

SUPPLEMENTARY MATERIALS

Supplementary Table 1: List of siRNA sequences used in the study

Gene Name	Sense	Antisense
E2F1	acgcuaugagaccucacug	cagugaggucucauagcgu
E2F2	uguaagcucaugcccuaga	ucuagggcaugagcuuaca
E2F3	ggugaccuguucucuagcu	agcuagagaacaggucacc
E2F7	cauuaagucgccaaacuau	auaguuuggcgacuuaaug
E2F8	gacaugccuaacacagcau	augcuguguuaggcauguc
p21	cuguacuguucugugucuu	aagacacagaacaguacag
NF-1C	cugguaucugggauagcaa	uugcuaucccagauaccag
CDP(CUTL1)	caaaaacagcacacucaaa	uuugagugugcuguuuuug
FAC1(BPTF)	gaggaaucuaaucucagua	uacugagauuagauuccuc
HMX1	caacacuaaacgucccuuu	aaagggacguuuaguguug
PBX1	ggaaauaagcgaauccggu	accggauucgcuuauuucc
DP1(TFDP1)	caggaaaacguuuagcgaa	uucgcuaaacguuuuccug

Supplementary Table 2: Primer sequences for qRT-PCR

Gene Name	Left Primer Sequence (5′→3′)	Right Primer Sequence (5′→3′)
CCNA1	aaggagtgtgcgtcaggact	accctgtaaatgcagcaagg
CCNA2	cctgcaaactgcaaagttga	aaaggcagctccagcaataa
CCNB1	catggtgcactttcctcctt	caggtgctgcataactggaa
CCND1	cgtggcctctaagatgaagg	ccacttgagcttgttcacca
CCND3	gcctcagccagagaagctat	aatgagccttcagcagcaaa
CCNE1	cccacagagacagcttggat	tctttggtggagaaggatgg
CDK2	tgtacctcccctggatgaag	catcctggaagaaagggtga
CDK4	ccgaagttcttctgcagtcc	acatctcgaggccagtcatc
CDK6	ttcggcctttcgcatacg	tgaaccaaaatgccacatac
P21	aagaccatgtggacctgtca	tctgtcatgctggtctgccg
P27	tgttagatagctgcatgtggcttt	gagtgatcaccattctgctgagtaa
P53	tcaacaagatgttttgccaactg	atgtgctgtgactgcttgtagatg
E2F1	tgcagagcagatggttatgg	ctcagggcacaggaaaacat
E2F2	gcagacgagactggaagtgc	tcctctgggcacaggtagac
E2F3	atatccctaaacccgcttcc	tgagcaatcgctatgtcctg
E2F4	gctgctggaggagttgatgt	ggtggagaaagacgaagcag
E2F5	gcagcagacatcagctacaga	gccttaagagaggaaacacgtc
E2F6	tcatctgagagcactcatcca	tctcaaatgccatcagttgc
E2F7	cacagtgtcgtccaacaacc	cttgggggtcactggaact
E2F8	gccaccaactatgactcacc	ctttgggtgtcacaggaacag
RPLP0	ccttgtctgtggagacggattac	gccaagaaggccttgacctt

Supplementary Table 3: List of antibodies used in western blotting

Antibody	Clone number	Company	Dilution
Cyclin A2	BF683	Cell signaling Technology	1/500
Cyclin B1	D5C10	Cell signaling Technology	1/500
Cyclin E1	HE12	Cell signaling Technology	1/500
p21Waf1/Cip1	12D1	Cell signaling Technology	1/500
p27Kip1	D69C12	Cell signaling Technology	1/500
p53	1C12	Cell signaling Technology	1/500
E2F8	ab109596	Abcam	1/500
α-tubulin	T6199	Sigma-Aldrich	1/1000
β-actin	13E5	Cell signaling Technology	1/1000

Supplementary Table 4: DAVID gene functional classification of genes upregulated by at least 1.5-fold by metformin in A549 cells

Keywords	Count	%	*P*-Value
Amino-acid biosynthesis	6	2.8	0.0000054
glycoprotein	71	32.6	0.00021
transmembrane protein	18	8.3	0.00075
Symport	7	3.2	0.0014
signal	53	24.3	0.0025
Apoptosis	12	5.5	0.0033
pyridoxal phosphate	5	2.3	0.0048
disulfide bond	46	21.1	0.01
iron	9	4.1	0.014
disease mutation	28	12.8	0.015
Aminotransferase	3	1.4	0.022
polymorphism	142	65.1	0.025
calcium	16	7.3	0.032
Serine biosynthesis	2	0.9	0.033
Immunoglobulin domain	11	5	0.035
Ichthyosis	3	1.4	0.043
heme	5	2.3	0.044
oxidoreductase	12	5.5	0.046
Secreted	27	12.4	0.049

Supplementary Table 5: Apoptosis-related genes upregulated by at least 1.5-fold by metformin in A549 cells

Gene Name	Gene Symbol	Log2 Ratio
BCL2-related protein A1	BCL2A1	0.6696
ChaC, cation transport regulator homolog 1 (E. coli)	CHAC1	1.2929
DNA-damage-inducible transcript 4	DDIT4	0.7288
SH3-domain kinase binding protein 1	SH3KBP1	0.7774
TSC22 domain family, member 3; GRAM domain containing 4	TSC22D3	0.6562
myocyte enhancer factor 2C	MEF2C	0.7269
phorbol-12-myristate-13-acetate-induced protein 1	PMAIP1	0.7991
similar to Rho-associated, coiled-coil containing protein kinase 1; Rho-associated, coiled-coil containing protein kinase 1	ROCK1	0.6312
similar to deubiquitinating enzyme 1; deubiquitinating enzyme 3; similar to deubiquitinating enzyme 3; ubiquitin specific peptidase 17	USP17, USP17L2	0.6556
tribbles homolog 3 (Drosophila)	TRIB3	1.3094
tumor protein p53 inducible nuclear protein 1	TP53INP1	0.705
tumor protein p63	TP63	0.7706

Supplementary Table 6: DAVID gene functional classification of downregulated (< 1.5 fold) genes by metformin in A549 cells

Keywords	Count	%	*P*-Value
cell cycle	23	12.8	1.80E–11
cell division	16	8.9	3.50E–09
mitosis	12	6.7	2.60E–07
lipid synthesis	7	3.9	1.40E–04
kinetochore	6	3.3	1.50E–04
kinase	16	8.9	4.30E–04
cell cycle control	4	2.2	1.90E–03
Steroid biosynthesis	4	2.2	4.00E–03
Fatty acid biosynthesis	4	2.2	4.90E–03
polymorphism	109	60.6	5.90E–03
steroid metabolism	4	2.2	6.70E–03
Cholesterol biosynthesis	3	1.7	1.00E–02
chromoprotein	4	2.2	1.30E–02
phosphoprotein	73	40.6	1.40E–02
transferase	20	11.1	1.60E–02
sterol biosynthesis	3	1.7	1.70E–02
metalloprotein	5	2.8	1.80E–02
atp-binding	19	10.6	1.90E–02
ubl conjugation	11	6.1	2.00E–02
fetus	2	1.1	2.40E–02
sugar transport	3	1.7	3.50E–02
ATP	6	3.3	4.20E–02

Supplementary Table 7: Cell cycle-related genes downregulated (< 1.5 fold) by metformin in A549 cells

Gene Name	Gene Symbol	Log2 Ratio
CDC28 protein kinase regulatory subunit 2	CKS2	–0.5916
E2F transcription factor 8	E2F8	–0.9603
NUF2, NDC80 kinetochore complex component, homolog (S. cerevisiae)	NUF2	–0.5976
SPC25, NDC80 kinetochore complex component, homolog (S. cerevisiae)	SPC25	–0.6377
anillin, actin binding protein	ANLN	–0.5823
antigen identified by monoclonal antibody Ki-67	MKI67	–0.593
asp (abnormal spindle) homolog, microcephaly associated (Drosophila)	ASPM	–0.5827
budding uninhibited by benzimidazoles 1 homolog (yeast)	BUB1	–0.6352
cell division cycle associated 3	CDCA3	–0.5999
claspin homolog (Xenopus laevis)	CLSPN	–0.6058
cyclin B3	CCNB3	–0.8182
cyclin D3	CCND3	–0.8234
cyclin F	CCNF	–0.5851
cyclin-dependent kinase inhibitor 3	CDKN3	–0.6387
discs, large (Drosophila) homolog-associated protein 5	DLGAP5	–0.7932
establishment of cohesion 1 homolog 2 (S. cerevisiae)	ESCO2	–0.5891
excision repair cross-complementing rodent repair deficiency, complementation group 6-like	ERCC6L	–0.6566
kinesin family member 11	KIF11	–0.6196
non-SMC condensin I complex, subunit H	NCAPH	–0.6131
polo-like kinase 1 (Drosophila)	PLK1	–0.6058
regulator of G-protein signaling 2, 24kDa	RGS2	–0.5867
sperm associated antigen 5	SPAG5	–0.729
ubiquitin-conjugating enzyme E2S	UBE2S	–0.6418

Supplementary Table 8: Putative transcription factors (TFs) responsible for mRNA reduction (< 1.5 fold) by metformin in A549 cells

Factor name	P-Value	Yes	No	Yes/No	Associated factors	Classification
MAZ	7.95128E-05	0.119	0.0123	9.7024	MAZ MAZ MAZ MAZ SAF1 SAF1	ZFC2H2
HNF-1alpha	0.00754763	0.0714	0.0123	5.8214	HNF-1alpha	HOX
NF-1	0.000986502	0.119	0.0245	4.8512	NF-1A NF-1C NF-1C NF-1C NF-1A NF-1A NF-1A1 NF-1A NF-1C NFI	SMAD
E2F	0.006505757	0.1071	0.0307	3.4929	E2F-1 E2F-1 E2F-1 E2F-1 E2F-3 E2F-3 E2F-4 DP-1 DP-1 E2F-7 E2F-3 E2F-4 E2F-1 E2F-4 DP-1 E2F-4 E2F-7 E2F-7 DP-1 E2F-1:DP-1 E2F:DP E2F:DP:E4	E2F
CDP CR1	0.003740592	0.125	0.0368	3.3958	CDP CDP CDP2 CDP	HOX
BRCA1:USF2	0.00067832	0.2202	0.0798	2.7614	brca1 brca1 brca1 brca1:USF2	ZFRING
REST	0.007293704	0.1548	0.0613	2.5226	REST REST REST REST REST	ZFC2H2
dlx-3	0.00049805	0.2917	0.1227	2.3771		HOX
TATA	0.007182177	0.2262	0.1104	2.0483	TBP TBP TBP TBP	TBP
ZFP105 secondary motif	0.009789789	0.2202	0.1104	1.9944	Zfp105	ZFC2H2
Pbx	0.005485282	0.2738	0.1411	1.9405	Pbx1 Pbx1 Pbx2 Pbx2 PBX1 PBX2 Pbx1 PBX3 Pbx3 Pbx1b:PREP-1 Pbx1:PREP-1 Pbx1:HOXB1 Pbx2:PREP-1 Pbx1a:HOXC6-isoform2 Pbx2:HOXC6-isoform2 PBX3a:HOXC6-isoform2 Pbx1a:HOXB7 Pbx1a:HOXB8 Pbx1a:ipf1 Pbx1a:Prep-1 Pbx1b:Prep-1 Pbx2:Prep-1 Pbx3a:Prep-1	HOX
HMX1	0.004497194	0.5476	0.3497	1.566	Homeobox protein H6 Homeobox protein H6	HOX
FAC1	0.003961633	0.7976	0.5521	1.4446	FAC1 FAC1 Falz	ZFPHD
HOXD12	3.28906E-06	2.3929	1.681	1.4235	HOXD12 HOXD12 HOXD12	HOX
Duxl	0.006883078	0.8869	0.6442	1.3768	Duxl	HOX
CDX-2	8.02777E-06	2.6845	1.9571	1.3717	Cdx-2 Cdx-2 Cdx-2 Cdx-3 Cdx-2 Cdx-2 Cdx-2 CDX-2	HOX
HOXB13	0.008657254	1.0833	0.8221	1.3178	HOXB13 HOXB13	HOX
Rhox11	0.007710806	1.2143	0.9325	1.3022	Rhox11	HOX
HOXC13	0.002059451	1.7619	1.362	1.2937	HOXC13 HOXC13	HOX
Irx2	0.004816385	1.5417	1.2025	1.2821	Irx2 Irx2	HOX
DRI1	0.00046911	2.5476	1.9939	1.2777	DRI1 DRIL1 DRI1	ARID
ZNF333	2.18785E-07	7.1786	5.7607	1.2461	Zinc finger protein 333	ZFC2H2
CRX	0.005762015	2.006	1.6258	1.2338	Crx Crx Crx crx	HOX
NF-AT1	0.009264913	3.5179	3.0429	1.1561	NF-AT1 NF-AT1 NF-AT1 NF-AT1 NF-AT1	REL

Supplementary Table 9: Clinicopathological characteristics (*N* = 848)

Variables	E2F8 expression		*P*-value
	Normal (N = 742)	Overexpressed (N = 106)	
Age (years)	66 ± 9	65 ± 10	0.05
Pack-years (smoking)	48 ± 29	43 ± 6	0.49
Diameter (cm)	1.2 ± 0.5	1.2 ± 0.4	0.50
Sex			
Men	421	68	
Women	321	38	0.15
Histology			
Adenocarcinoma	417	58	
Squamous cell carcinoma	325	48	0.77
Pathologic stage			
I	387	50	
II	212	26	
III	114	23	
IV	20	4	0.28
Recurrence			
No	473	60	
Yes	269	46	0.15

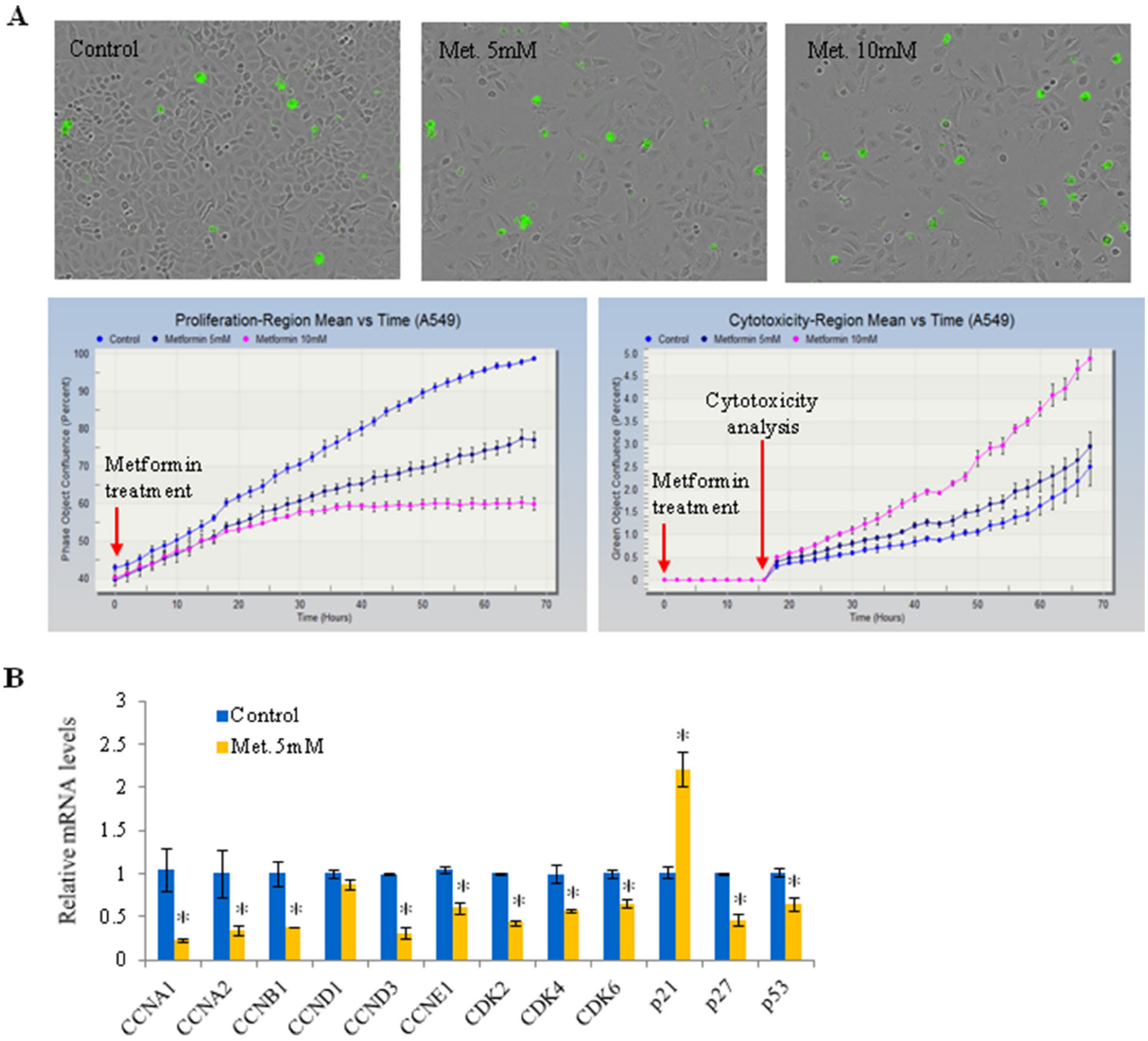

Supplementary Figure 1: The effect of metformin on the expression of cell cycle-related genes in A549 lung cancer cells. (**A**) A549 cells were treated with metformin (5 mM, 10 mM). The upper panels show representative images of cultured A549 cells. Green indicates dead cells stained with cyanine dye. The lower panels show percentage of confluence (left) and percentage of cytotoxicity (right). The images were obtained every two hours, and cyanine dye was added 16 h after metformin treatment. (**B**) A549 cells were treated with 5 mM metformin and the mRNA levels of cell cycle-related genes were measured by qRT-PCR. The mRNA level in each sample was normalized to the internal control, RPLP0. Values on the y-axis indicate fold change in mRNA level compared to the control (n = 3, $^{*}P$ = 0.05). The "Met." indicates metformin.

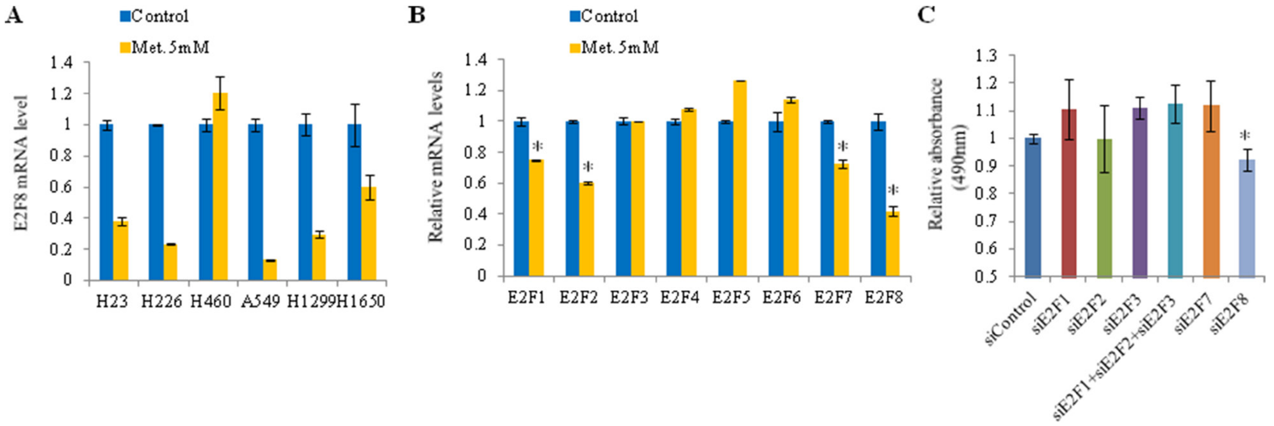

Supplementary Figure 2: The effect of metformin on the expression of E2F8 family members in A549 lung cancer cells. (**A**) H23, H226, H460, A549, H1299, and H1650 lung cancer cells were treated with 5 mM metformin, and E2F8 mRNA levels were measured by qRT-PCR. (**B**) A549 cells were treated with 5 mM metformin, and the mRNA levels of the E2F family members were measured by qRT-PCR ($n = 3$, $^{*}P < 0.05$). (**C**) A549 cells were transfected with the indicated siRNAs, and cell proliferation was detected by the MTS assay on the third day after transfection ($n = 8$, $^{*}P < 0.05$). The "Met." indicates metformin.

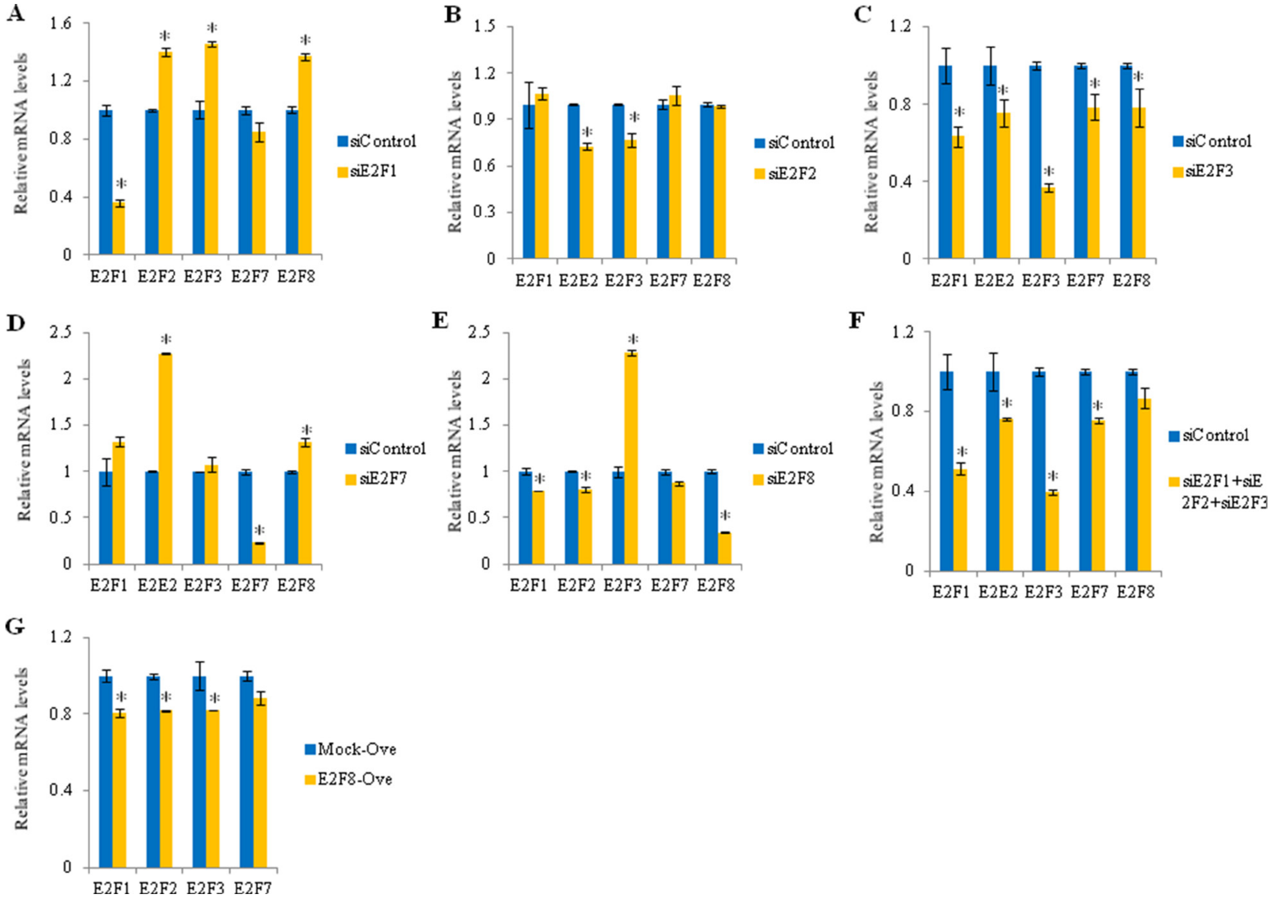

Supplementary Figure 3: The effect of E2F family member knockdown or E2F8 overexpression on the expression of other E2F family members. (**A–F**) H1299 cells were transfected with siRNAs directed against E2F1 (A), E2F2 (B), E2F3 (C), E2F7 (D), E2F8 (E), or with a combination of E2F1, E2F2, and E2F3 siRNAs (F), then the mRNA level of these genes were detected by qRT-PCR. (G) In addition, the effects of E2F8 overexpression on the expression of other E2Fs were analyzed using qRT-PCR. The mRNA levels were normalized to RPLP0, and the values on the y-axis represent fold change in mRNA level relative to control ($n = 3$, $^{*}P < 0.05$). The "Ove" indicates overexpression.

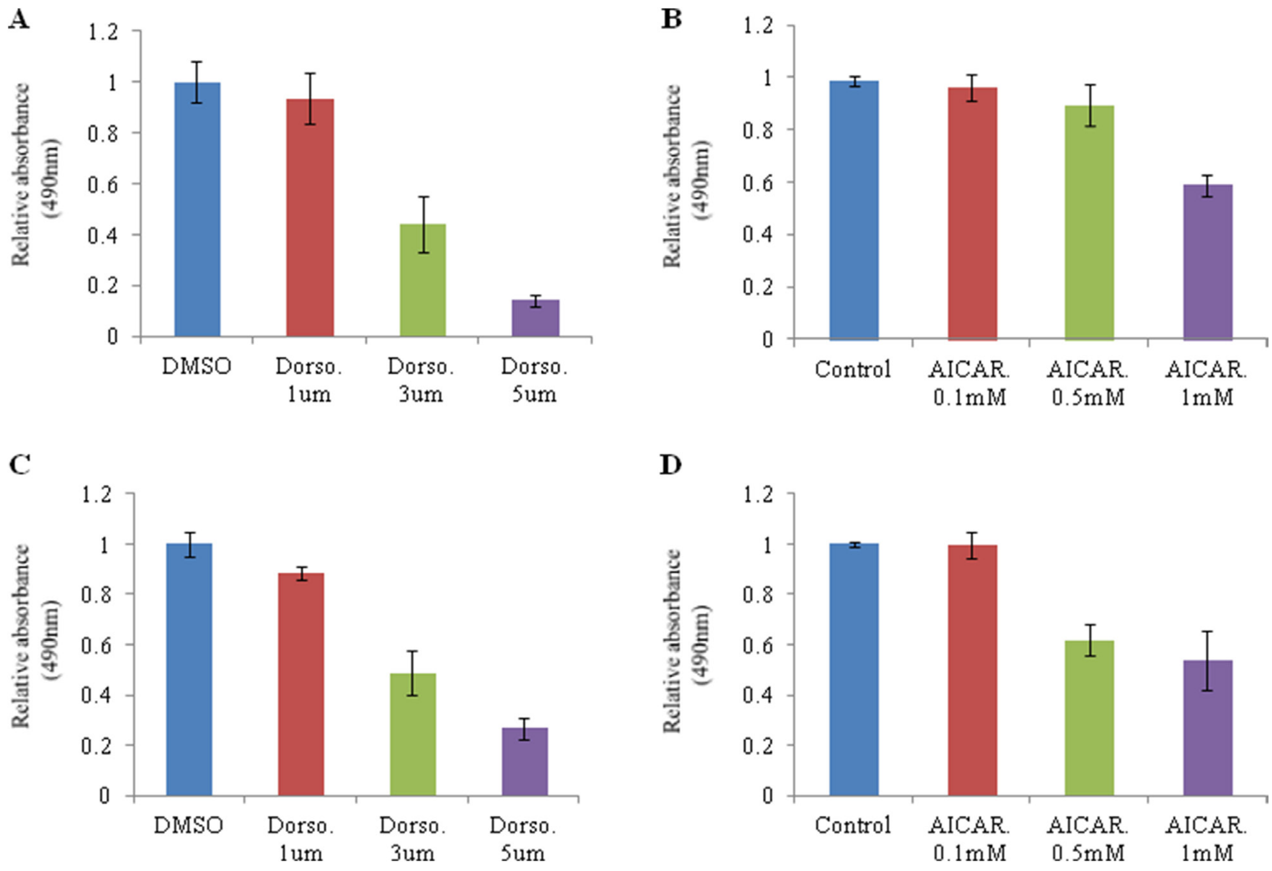

Supplementary Figure 4: The effect of Dorsomorphin and AICAR on cell proliferation. A549 (**A**, **B**) and H1299 (**C**, **D**) cells were treated with Dorsomorphin or AICAR. Cell proliferation was detected by MTS assay. Error bars indicate standard deviation (n = 8, $^{*}P < 0.05$).
